# Injury Profile of Male and Female Senior and Youth Handball Players: A Systematic Review

**DOI:** 10.3390/ijerph17113925

**Published:** 2020-06-01

**Authors:** Javier Raya-González, Filipe Manuel Clemente, Marco Beato, Daniel Castillo

**Affiliations:** 1 Facultad de Ciencias de la Salud, Universidad Isabel I, 09001 Burgos, Spain; rayagonzalezjavier@gmail.com; 2 Escola Superior Desporto e Lazer, Instituto Politécnico de Viana do Castelo, Rua Escola Industrial e Comercial de Nun’Álvares, 4900-347 Viana do Castelo, Portugal; filipe.clemente5@gmail.com; 3 School of Health and Sports Science, University of Suffolk, Ipswich IP4 1QJ, UK; M.Beato@uos.ac.uk

**Keywords:** incidence, risk factors, team sport

## Abstract

Handball is a team sport in which players are exposed to high physical conditioning requirements and several contacts and collisions, so they must face various musculoskeletal injuries throughout their career. The aim of this study was to summarize the characteristics of handball injuries both in training and in competition contexts, differentiating by gender and age. A systematic review was conducted and a total of 15 studies (33 cohorts) met the inclusion criteria. Higher injury incidence was reported during matches compared to training sessions in all groups (i.e., male and female senior and youth players), with male senior players presenting the greatest values. Lower extremities were more frequently injured, being contusions and sprains the most common type of injuries. Females reported more serious injuries than males, who presented a higher percentage of acute injuries caused by direct contact, while in female players these injuries were not caused by direct contact actions. Wings and backs presented the highest injury incidence; additionally, players registered higher match incidence during international championships compared to national leagues. Due to the differences in the injury profile of handball players, specific preventive strategies should be implemented for each group to optimize the injury prevention process.

## 1. Introduction

Handball is a team sport founded in 1946 and included in the Olympic Games list for the first time in 1972 [1]. The popularity of this sport has increased in recent years, and currently, there are an estimated 25 million players worldwide [2], including male, female, senior, and youth players [3]. Despite the multiple beneficial effects derived from handball practice, such as improvements in cardiovascular, metabolic, muscular and psychosocial health [4,5,6], this team sport presents a high injury risk [7], mainly due to its high-intensity specific demands (i.e., rapid changes of direction, jumps with abrupt landings and repetitive throws, as well as frequent physical contact among players [8,9]). Likewise, low physical fitness, incorrect technique, lack of flexibility, and also inadequate rehabilitation treatment of injuries have been reported as risk factors related to the occurrence of injuries [10,11]. Additionally, the high training volumes and intensities that youth players undertake to achieve sporting excellence seem to contribute to the increase of the injury incidence [10]. In this regard, injuries are associated with negative consequences, such as a reduction of team success [12], an increment of costs related to treatments [13], and the risk of suffering new injuries [14]. In addition, injuries might also have long-term health consequences influencing handball players’ quality of life and career [15]. Therefore, reducing the injury incidence can have a key positive impact for both players’ and teams’ performance.

To comprehensively address this issue, it seems necessary to apply a structured injury prevention approach [16]. In this sense, Van Mechelen et al. [17] established that epidemiological analysis must be the first step in developing effective injury prevention strategies, incorporating not only information about injury incidence (i.e., likelihood), but also burden and availability (i.e., consequence) values [18]. Regarding this, several studies have analyzed handball players’ injury profile, showing an overall incidence of 4.1–12.4 injuries/1000 h overall exposure [19,20,21]. Likewise, training incidence in handball players is established between 0.6 and 4.6 injuries/1000 h training [3,19,22], and match incidence is set at 10.8–73.6 injuries/1000 h [1,3,19], confirming that match incidence is significantly higher than training incidence. Additionally, the lower extremities seem to be the body area where most injuries are sustained, affecting mainly the ankle, knee and head, with ligament sprains and muscle strains being the most frequent type of injuries [19,20,23]. Despite the great number of epidemiological studies focused on handball players, there is a discrepancy of definitions addressing injuries and data collection procedures, which suggests a need to perform a detailed study of the injury profile in handball with the aim of expanding and clarifying the current knowledge regarding handball injuries.

Injuries are considered a complex phenomenon [24] produced by the interaction of multiple risk factors [14], where players’ characteristics (e.g., gender or age) are the most influential ones [25]. In this sense, previous studies have reported that injury risk increases with age or according to the gender of the players (i.e., higher injury risk for male players) [26], which may be explained by different game behaviors and physical contact among players [21,27]. Additionally, training load performed by the players during matches and training sessions should be taken into account when injury risk factors are analyzed [14]. Regarding this, previous studies have shown that training and match loads in terms of distance covered at high intensities are greater in male players compared to female ones [28] and between senior and youth [29] handball players; thus, these variables should be investigated within the analysis of the injury profile in cohort studies. In this sense, several epidemiological studies have focused on senior male and female players [3,19,22,30], and others on youth male and female handball players [3,20,21,31]. However, despite the key effect of gender and age on injuries in team sports [32,33,34,35], to date, no systematic reviews have been carried out to expand the knowledge about the injury profile (considering injury incidence, location, severity and type) in handball players.

Despite the increased interest in injuries associated with handball practice, no definitive evidence currently exists, and it is necessary to conduct a systematic review to generate robust conclusions about the injuries that take place in this sport and consequently facilitate their prevention process. Therefore, the objective of this systematic review is to summarize the characteristics of handball injuries in both training and competition, differentiating between gender and age.

## 2. Materials and Methods

The present review was carried out following the recommendations and criteria established in the Preferred Reporting Items for Systematic Reviews and Meta-analysis (PRISMA) statement guidelines [36].

### 2.1. Search Strategy

For this systematic review, potential studies were identified in PubMed/MEDLINE, SPORTDiscus, and Web of Science (including all Web of Science Core Collection: Citation Indexes) databases. The search syntax included the following keywords coupled with Boolean operators: “handball” AND (“injury” OR “injuries” OR “epidemiology” OR “prevalence” OR “incidence”). A year restriction was applied for this search (i.e., studies published between 2000 and 2019). Additionally, a secondary search was performed based on the screening of the reference lists these studies and the studies that cited the included studies through Google Scholar. Two authors (JRG and DC) independently screened the title and abstract of each reference to locate potentially relevant studies and reviewed them in detail to identify articles that met the inclusion criteria. Any discrepancies between the authors in the selection process were solved in consultation with a third reviewer (FMC).

### 2.2. Inclusion Criteria

The studies included in the present review had to fulfil the following inclusion criteria: (1) the sample must be composed only of handball players, (2) studies that analyzed the injury profile of different groups must report the data in a differentiated way (i.e., specific data of each group), (3) studies must report injury incidence or provide sufficient data to calculate it through standardized equations, (4) studies that reported values of time-loss injuries or allow the possibility to calculate it, and (5) studies had to be the full-text published in a peer-reviewed journal. In addition, conference abstracts, letters to the editor, errata, narrative reviews, systematic reviews, meta-analyses or invited commentaries and studies that were not written in English were also excluded.

### 2.3. Study Coding and Data Extraction

The following moderator variables were extracted from the included studies: (a) authors, year of publication and study design, (b) sample characteristics (including sample size, age, region and status), (c) follow-up duration, and (d) epidemiological data (including incidence, exposure, severity, burden, type of injury, location, and playing position).

### 2.4. Methodological Quality Assessment

The methodological quality of the included studies was assessed using a risk of-bias quality form of 15 items validated and adjusted for the specific context of epidemiological research [37], to provide guidance to facilitate a critical appraisal and interpretation of the results. Each question was answered with yes if the criteria were satisfied (2 points), with don’t know (1 point), or with a no if the criteria were not satisfied (0 points). All 15 quality criteria are presented online as Supplementary material (i.e., Table S1). Based on this procedure, the studies were classified as follows: low methodological quality (≤50% of total points); good methodological quality (51–75% of total points); and excellent methodological quality (>75% of total points).

Data extraction and methodological quality assessment were performed independently by two authors (JRG and DC) and discrepancies between the authors were resolved in consultation with a third reviewer (FMC). To assess the reliability of the process, intraclass correlation coefficient (ICC) and the Cohen’s κ coefficient were calculated showing an ICC of 0.94 (0.84–1.0) and a κ coefficient of 0.92 (0.83–1.0).

## 3. Results

### 3.1. Search Results

**Figure 1:**
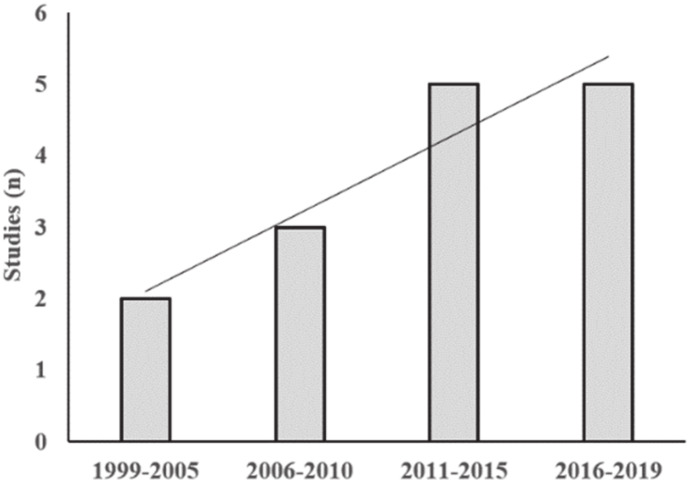
Date of publication of the selected studies.

**Figure 2:**
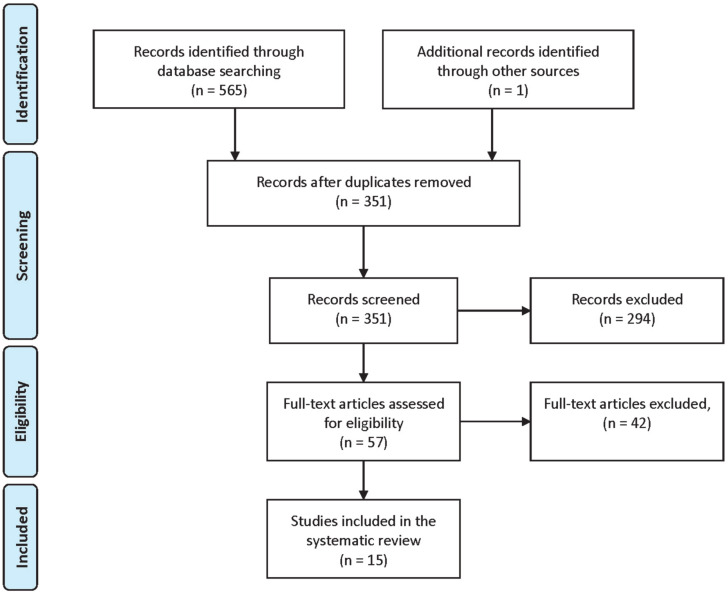
Flow diagram of the study retrieval process.

Figure 1 shows the evolution of the studies published on this topic for every 5-year period along the last 20 years, while the flow diagram of the study retrieval process performed in this research is reported in Figure 2.

### 3.2. Descriptive Characteristics of the Studies

The included studies are summarized in Table 1, Table 2, Table 3 and Table 4. The selected 15 studies resulted in 33 cohorts, as nine studies had more than one group. Eleven studies were carried out with senior male handball players [1,3,19,20,22,23,30,38,39,40,41], five with senior female handball players [3,22,30,39,42], five with youth male handball players [3,20,21,31,40], and four with youth female handball players [3,21,31,43]. These studies were carried out between 1999 and 2019 and comprised a total of 12,687 participants, divided as follows: 3516 senior male handball players, 952 senior female handball players, 4330 youth male handball players and 3889 youth female handball players. In addition, 12 studies [3,19,20,21,22,23,30,38,39,40,42,43] used a prospective cohort design, while the remaining three studies [1,31,41] used a retrospective cohort design. Finally, the identified studies had a duration between one month and six seasons.

#### 3.2.1. Injury Incidence: Overall, Training and Match

Eleven studies (24 cohorts) reported information about the overall injury incidence [1,3,19,20,21,22,23,40,41,42,43], while fifteen studies (27 cohorts) reported match injury incidence [1,3,19,20,21,22,23,30,31,38,39,41,43], and nine studies (18 cohorts) reported training injury incidence [1,3,19,20,22,23,41,43].

According to the senior groups, eight studies reported overall incidence [1,3,19,20,22,23,40,41], ten reported match incidence [1,3,19,20,22,23,30,38,39,41], and six reported training incidence [1,3,19,22,23,38] in male handball players, while three studies reported overall incidence [3,22,42], four reported match incidence [3,22,30,39] and two reported training incidence [3,22] in female handball players. With regard to youth groups, four studies reported overall incidence [3,20,21,40], four reported match incidence [3,20,21,31], and three reported training incidence [3,20,21] in male handball players; and three studies reported overall incidence [3,21,43], four reported match incidence [3,21,31,43], and three reported training incidence [3,21,43] in female handball players.

#### 3.2.2. Location and Type of Injuries

Injury location was reported in eight studies (14 cohorts) [1,19,22,23,30,31,38,43] distributed as follow: six studies in senior male handball players [1,19,22,23,30,38], two studies in senior female handball players [22,30], one study in youth male handball players [31], and two studies in youth female handball players [31,43].

Regarding the type of injuries, five studies (11 cohorts) reported this information [1,22,30,31,44], four studies in senior male handball players [1,19,22,30], three studies in senior female handball players [22,30,42], one study in youth male handball players [31], and one study in youth female handball players [31].

#### 3.2.3. Severity and Mechanism

Nine studies (19 cohorts) reported the severity of injuries. As regards to this matter, eight studies reported the severity in senior male handball players [1,19,22,23,30,39,40,41], three studies in senior female handball players [22,30,39], one study in youth male handball players [40], and one study in youth female handball players [43].

Injury mechanism was reported in seven studies (13 cohorts) [19,21,22,23,30,39,41] such as: six studies in senior male handball players [19,22,23,30,39,41], three studies in senior female handball players [22,30,39], one study in youth male handball players [21], and one study in youth female handball players [21].

#### 3.2.4. Playing Position and Competition

A total of eight studies (11 cohorts) reported information of injuries differentiating between playing positions (i.e., goalkeeper, back, wing and line) [19,20,22,23,31,38,41,43]. In this regard, six studies reported the severity in senior male handball players [19,20,22,23,38,41], one study in senior female handball players [22], two studies in youth male handball players [20,31], and two studies in youth female handball players [31,43].

Attending to the competition type, eleven studies (25 cohorts) were performed during national leagues while four studies (8 cohorts) reported injury information related to international championships (i.e., Olympic games, World championship and Europe championship).

### 3.3. Methodological Quality Assessment

Table S1 shows the individual scores for the quality assessment. Values ranged from 22 to 28 points, with an average score of 25 points. Regarding the individual quality assessment, thirteen studies were categorized as excellent, while the two remaining studies were categorized as being of good quality.

## 4. Discussion

The aim of this systematic review was to analyze the injuries derived from handball practice in both training sessions and matches, differentiating by gender and age. Despite the growing interest in injures in handball [23], this is the first systematic review that summarizes the injury profile of handball players according to gender (i.e., male and female) and age (i.e., senior and youth). This knowledge could provide valuable information for detecting possible factors associated with injuries in different groups of handball players, aiming to facilitate the implementation of specific preventive strategies.

### 4.1. Injury Incidence: Overall, Training and Match

A key variable for understanding the impact of injuries on athletes is the incidence (i.e., number of injuries/1000 h exposure) [45]. In this sense, the handball players included in our systematic review presented values ranging between 1.7 and 7.8 injuries/1000 h exposure. Specifically, senior male handball players showed the highest value (i.e., near to 7.8 injuries/h exposure), while lower incidences were observed in female senior players (i.e., 6.2 injuries/h exposure), in male youth players (i.e., 6.9 injuries/h exposure), and in female youth players (i.e., 6.8 injuries/h exposure). These differences could be due in part to high-intensity and faster play speed reported during male senior handball practice [3,21]. According to this, differences between categories are accentuated when match incidence is analyzed. In this sense, senior male players match incidence range from 15 to 73.6 injuries/h match exposure, which are the greatest values compared to female senior players (i.e., 13–36 injuries/h exposure), male youth players (i.e., 14.9–32.7 injuries/h exposure) and female youth players (i.e., 10.8–23.8 injuries/h exposure). Although higher match incidence was highlighted for senior male players, similar training incidence was observed in all categories (i.e., between 0.96 and 4.1 injuries/1000 h training exposure). These reported values show that training incidence is substantially lower in comparison to match incidence in all groups, in line with those studies focuses on other team sports (e.g., soccer [32] or basketball [35]). These differences may be associated with several factors, for instance, the higher physical and physiological demands performed by players during matches compared to training sessions [46], the variability and uncertainly of the game, as well as the neuromuscular and mental fatigue generated during matches [47], or because of different training load quantification and periodization strategies [48]. Therefore, strength and conditioning coaches should focus on recreating the physical, technical, tactical, and psychological demands of competition during the training sessions, as well as implementing specific recovery strategies to reduce the negative impact of the matches (e.g., accumulated fatigue) on the handball players (e.g., excessive fatigue or uncertainly), and consequently, to reduce the injury risk [46].

### 4.2. Location and Type of Injuries

**Figure 3:**
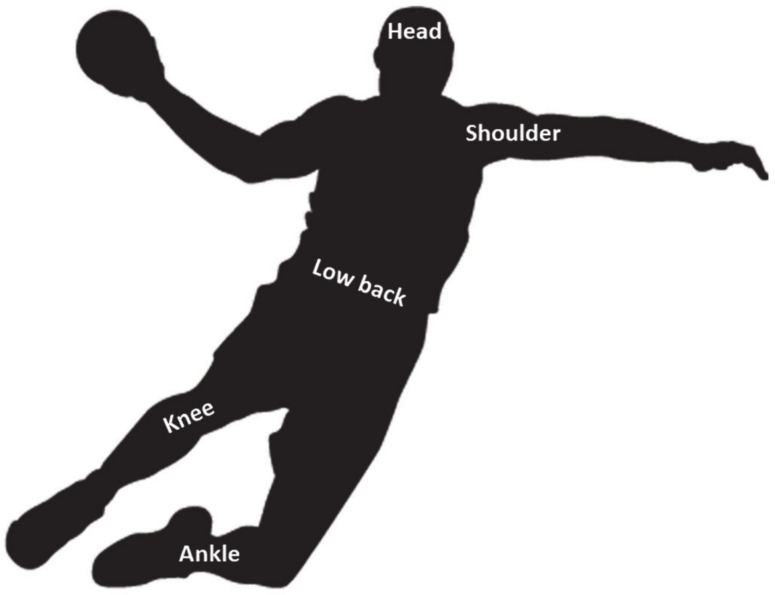
Locations with increased risk of injury.

From a practical point of view, it is crucial to understand the injury locations to make effective decisions during the injury prevention process (Figure 3). In this regard, an overall analysis of the studies included in this systematic review revealed that the most common injured areas in handball players (considering all categories) were the lower limbs, representing between 40% and 77% of the total injuries [1,19,22,23,30,31,38,43]. This could be explained by the changes in game rules during recent years, which have made the rules regarding contact between players more restrictive (e.g., trunk use instead the body to block the opponent or prohibition of dangerous elbow use both as a starting position and when in motion). This fact has led to a reduction in high-intensity bumps, contacts and collisions that would previously have resulted in more frequent upper limb injuries [19]. Specifically, the knee and the ankle seem to be the most damaged areas (i.e., near to 20% in each of the two locations), due to the implication of these joints in specific patterns of the most common actions in handball (e.g., jumps, decelerations or landings). Nevertheless, some authors [22] have shown a great incidence of overuse injuries in the shoulders (44%), caused by the repetitive throwing gesture imposed in this sport. Likewise, low back overuse injuries presented a high injury incidence (39%), possibly due to the extreme actions related to collisions and landings [49]. With respect to gender-related differences, Giroto et al. [22] observed a greater number of knee injuries in female senior handball players compared to their male partners (i.e., 38 vs. 14 injuries) during one season follow-up. This could be based on the reported gender differences in knee anatomy [50] and in proximal control and kinematics variables during common handball tasks, such as running or landing [51,52]. These differences in knee injuries have not been reported in youth handball players, since maturational changes take place at these ages [53]. However, male youth players showed a higher incidence in head/face and shoulder injuries in comparison with their youth female counterparts (35 vs. 18), possibly due to the more aggressive behavior and more frequent contact between players observed in this population [21,27]. It could be interesting to perform future research studying the relationship between playing positions and injury location.

With respect to the type of injury, similar patterns were observed between male and female senior handball players, with contusions and sprains being the most common. Nonetheless, studies referring to earlier championships (i.e., 2001–2003) [30] observed a higher prevalence of contusion injuries (i.e., near to 50%), possibly due to the rules changes mentioned above not having been implemented in those championships. On the other hand, Giroto et al. [22] reported that strains were the most common type of injury in male senior handball players (32.4%), perhaps influenced by the high physical demands during handball practice [28]. Additionally, in this study, also training injuries were analyzed, a fact that could underpin these results. Attending to youth populations, Asai et al. [31] showed a higher incidence in sprain injuries (128 injuries), with very high values compared to contusions (80 injuries). These differences with senior players could be due to youth players still not having a fully developed musculoskeletal system [54]. However, this finding should be taken with caution because of the lack of studies reporting injury type in youth handball players [31]. In this sense, further research investigating youth handball players, including information about injury location and type, is necessary to understand injury etiology and, subsequently, to propose specific preventive protocols for these populations.

### 4.3. Severity and Mechanism

Although injury incidence has been used as a quantitative parameter to analyze the impact of injuries [55], consequences of injuries should be assessed through the severity parameter to better understand their real impact [18]. In this sense, injuries with a duration of less than 7 days (i.e., 1–7 absence days) are the most commons in this systematic review. However, this evidence is relatively weak, because not all studies used the same criteria to classify injuries according to their severity. Specifically, in male handball players, including senior and youth, injuries of 1–7 absence days were reported as the most common, presenting values near to 65% of overall injuries [1,22,40]. Nevertheless, when international championships were analyzed (i.e., only values of match injuries are reported during congested periods) most injuries (near to 50%) resulted in 1–3 days of absence [19,30,39]. Despite male and female players showing similar results, a tendency to suffer more serious injuries (i.e., 7–28 absence days) was observed in senior and youth female handball players [22,43]. These findings seem to be imprecise, due to the aforementioned discrepancy with the severity classification; thus, it would be appropriate to present the value of burden (i.e., number of absence days/1000 h exposure) [18]. Regrettably, these studies only report incidence and severity, with the average number of absence days not being considered, so it was not possible to calculate the burden. Additionally, the availability (i.e., Σ of player match opportunities (number of team matches x squad size) —Σ of player match absences due to injury) is considered to be a new key indicator in sports injury research [12], although no data have been included in the selected studies. Therefore, further research assessing injuries through three different prisms (i.e., incidence, burden and availability) is necessary to help coaches to understand the meaningfulness of injury episodes in handball players across all ages, and thus to optimize the application of preventive programs.

Regarding injury mechanisms, acute injuries (i.e., those resulting from a specific and identifiable event) seem to be the most common in all the analyzed categories compared to overuse injuries (i.e., those caused by repeated micro-trauma without a single, identifiable event responsible for the injury), presenting values 55% to 85% of overall injuries [19,22,23,30,39]. These values are similar when comparing male and female handball players, although a higher percentage of acute injuries caused for a direct contact (e.g., collisions) were reported in male players, while female players suffer more acute injuries by no direct contact actions (e.g., landings) [22]. On the other hand, Piry et al. [41] reported specific mechanisms and observed that the most risky actions for these male senior handball players were plants and cuttings (28.57%), following of blocking (22.22%), shooting (20.63%), and turning (19.05%). With respect to youth handball players, only one study was included in our systematic review [21]. These authors reported similar values in male and female youth players, which were in line with values observed in senior players, with the acute injuries as the most common (75–80% of overall injuries). Even though acute injuries are difficult to prevent since they are mainly caused by collisions with teammates or opponents, instead it has been shown that neuromuscular training can reduce the incidence of overuse and non-contact injuries, as well as the burden derived from them [49].

### 4.4. Playing Position and Competition

The heterogeneity of criteria observed when classifying handball players according to playing position makes the comparison among studies difficult. In this regard, some authors only differentiated between goalkeepers and outfield players [31], other authors divided the players into goalkeepers, first line (i.e., backs and center backs) and second line (i.e., wing and line players) [20], while most studies classified players by specific playing positions (i.e., goalkeeper, backs, wing, and line) [19,22,23,38,41,43]. In spite of this limitation, the results observed in the included studies indicate that outfield players reported more injuries than goalkeepers in all the analyzed categories, ranging from 88% to 95%. Specifically, back [38] and wing [19,43] were the playing positions which presented the highest injury incidence, since each handball position is characterized by different tasks during practice [8]. However, this data must be taken with caution, because the majority of the studies reported the injuries in absolute values (i.e., percentage of total injuries); to clarify this point it is necessary to know the injury incidence (i.e., number of injuries/1000 h exposure) and the burden (i.e., number of absence days/1000 h exposure) to understand the magnitude of the injury pattern for each playing position. With respect to the type of exposure, Mónaco et al. [20] reported in male senior and youth handball players that first line players suffered a higher incidence during training, while the injury incidence during matches was greater in second line players. Although further studies focused on the injury incidence of each playing position are necessary, this information provides a novel knowledge to improve the implementation of specific preventive programs in handball players.

Handball competitions present different characteristics (e.g., play-off, congested schedule or use of players of the reserve team) that can influence the injury incidence of the players [56]. Therefore, it seems pertinent to analyze whether there are differences in the incidence during matches when the national league or international championships are played. In this regard, studies based on international championships showed higher injury incidence (from 30.9 to 50.5 injuries/1000 h match) in comparison to national leagues (from 15 to 31.7 injuries/1000 h match). These differences suggest the necessity of implementing specific injury prevention, load monitoring and recovery strategies to try to reduce the injury risk during international championships. Additionally, future studies should be performed in order to know the training incidence during the international championships to optimize the injury prevention process.

### 4.5. Limitations

This study is not exempt of limitations. Firstly, differences in classification of several variables such as severity or playing position complicate the comparisons among studies. In addition, none of the included studies reported injury incidence related to all variables, instead, they presented absolute and percentage values. Secondly, available literature related to youth handball players is scarce, especially for some variables such as injury mechanism, severity and playing positions. Finally, none of the included studies reported information regarding burden, absence days and availability, information that would improve the strength of this systematic review. On the other hand, the main value of this study is that allows to establish for the first time an overall evidence of incidence in handball, differentiating by age and gender, which are factors associated with injuries. This review is a key step forward for the development of specific preventive programs with handball players.

### 4.6. Practical Applications

In a practical approach, the findings observed in the present systematic review will make it possible to perform specific preventive programs attending to age and gender in handball players. In this respect, preventive programs should focus mainly on the riskiest locations and in the most prevalent type of injury for each group. Additionally, these programs should attend to the needs of each playing position and try to reproduce the most frequent injury mechanisms. Finally, due to the higher match incidence in all groups, training sessions should recreate the physical, technical, tactical, and psychological demands of competition in order to reduce the injury risk.

## 5. Conclusions

Handball players presented a higher injury incidence during matches than during training, with the male senior players having the highest overall values of training and match incidence. The lower extremities were the most commonly injured areas, with particular emphasis on the ankle and the knee for male players, and especially knee injuries in female players. Contusions and sprains were the most common type of injuries in senior female and youth handball players, while strains had a great incidence in male handball players. Injuries lasting fewer than 7 days were the most common in all the analyzed groups, although female players reported more serious injuries (i.e., 7–28 absence days). Acute injuries were more frequent than overuse ones, even though male players suffered a higher percentage of acute injuries caused by direct contact, while female players reported more acute injuries without contact. Regarding the playing position, wings and backs presented the highest percentages of injuries among playing positions. Finally, match injury incidence was higher during international championships compared to national leagues. All the included studies were categorized as having a good or excellent methodological quality, which therefore strengthens the conclusions of this systematic review.

## Figures and Tables

**Table 1:** Group and intervention characteristics in senior male handball players.

Study	Study Design	Age Range	Number of Participants (N)	Region	Status	Duration
Bere et al. (2015) [19]	Prospective cohort study	N/D	384	International	Elite	World championship
Giroto et al. (2015) [22]	Prospective cohort study	24.1 y	156	Brazil	Elite	One season
Junge et al. (2006) [43]	Prospective cohort study	N/D	168	International	Elite	Olympic games
Langevoort et al. (2006) [30]	Prospective cohort study	N/D	384	International	Elite	World championship
Langevoort et al. (2006) [30]	Prospective cohort study	N/D	384	International	Elite	World championship
Luig et al. (2018) [1]	Retrospective cohort study	25.8 y	549	Germany	Elite	Three seasons (First division)
Luig et al. (2018) [1]	Retrospective cohort study	24.8 y	828	Germany	Elite	Three seasons (Second division)
Moller et al. (2012) [3]	Prospective cohort study	24.9 y	56	Denmark	National level	31 weeks
Mónaco et al. (2013) [38]	Prospective cohort study	28.3 y	89	Spain	National level (First team)	Three seasons
Mónaco et al. (2013) [38]	Prospective cohort study	20.1 y	79	Spain	National level (Second team)	Three seasons
Mónaco et al. (2019) [20]	Prospective cohort study	20.4 y	31	Spain	National level (Second team)	Two seasons
Piry et al. (2011) [40]	Retrospective cohort study	N/D	40	Asia	Asian level	One year
Rafnsson et al. (2017) [23]	Prospective cohort study	23.6	109	Iceland	National level	One season
Tabben et al. (2019) [41]	Prospective cohort study	N/D	387	International	Elite	World championship

N/D: non data reported; y: years.

**Table 2:** Group and intervention characteristics in senior female handball players.

Study	Study Design	Age Range	Number of Participants (N)	Region	Status	Duration
Giroto et al. (2015) [22]	Prospective cohort study	22.8 y	183	Brazil	Elite	One season
Junge et al. (2006) [43]	Prospective cohort study	N/D	168	International	Elite	Olympic games
Langevoort et al. (2006) [30]	Prospective cohort study	N/D	384	International	Elite	World championship
Langevoort et al. (2006) [30]	Prospective cohort study	N/D	256	Europe	Elite	Europe Cup
Moller et al. (2012) [3]	Prospective cohort study	23.2	75	Denmark	National level	31 weeks
Petersen et al. (2005) [39]	Prospective cohort study	N/D	142	Germany	National level	One season

y: years; N/D: non data reported.

**Table 3:** Group and intervention characteristics in youth male handball players.

Study	Study Design	Age Range	Number of Participants (N)	Region	Status	Duration
Asai et al. (2019) [31]	Retrospective cohort study	13–14 y	3780	Japan	National level	Six seasons
Moller et al. (2012) [3]	Prospective cohort study	17.6 y (U18)	41	Denmark	National level	31 weeks
Moller et al. (2012) [3]	Prospective cohort study	15.7 y (U16)	28	Denmark	National level	31 weeks
Mónaco et al. (2013) [38]	Prospective cohort study	16.1 (U17)	85	Spain	National level	Three seasons
Mónaco et al. (2013) [38]	Prospective cohort study	14.7 y (U15)	87	Spain	National level	Three seasons
Mónaco et al. (2013) [38]	Prospective cohort study	12.7 y (U13)	69	Spain	National level	Three seasons
Mónaco et al. (2019) [20]	Prospective cohort study	14.4 y (U15)	133	Spain	National level	Two seasons
Olsen et al. (2006) [21]	Prospective cohort study	U17	107	Norway	National level	Seven months

y: years; U: under.

**Table 4:** Group and intervention characteristics in youth female handball players.

Study	Study Design	Age Range	Number of Participants (N)	Region	Status	Duration
Asai et al. (2019) [31]	Retrospective cohort study	13–14 y	3300	Japan	National level	Six seasons
Moller et al. (2012) [3]	Prospective cohort study	17.5 y (U18)	53	Denmark	National level	31 weeks
Moller et al. (2012) [3]	Prospective cohort study	15.7 y (U16)	89	Denmark	National level	31 weeks
Olsen et al. (2006) [21]	Prospective cohort study	U17	321	Norway	National level	Seven months
Wedderkopp et al. (1999) [42]	Prospective cohort study	16–18 y	126	Europe	Elite	One season

y: years; U: under.

## Supplementary Materials

The following is available online at https://www.mdpi.com/1660-4601/17/11/3925/s1, Table S1: Methodologic quality of the included studies for handball players.
