# Supplementary material for: Injury Profile of Male and Female Senior and Youth Handball Players: A Systematic Review
Source: Int J Environ Res Public Health. 2020 Jun 1;17(11):3925. doi: 10.3390/ijerph17113925 (PMC7312653; doi:10.3390/ijerph17113925)
Supplement: Supplementary file 1 [file ijerph-17-03925-s001.pdf]

Table 1. Methodologic quality of the included studies for handball players.

Study	1	2	3	4	5	6	7	8	9	10	11	12	13	14	15	Score	Quality
Asai et al. (2019) [**31**]	yes	yes	no	yes	yes	yes	no	yes	no	yes	yes	yes	yes	yes	no	22	Good
Bere et al. (2015) [19]	yes	yes	no	yes	yes	yes	yes	yes	yes	no	yes	yes	yes	yes	yes	26	Excellent
Giroto et al. (2015) [22]	yes	yes	no	yes	yes	yes	yes	yes	yes	yes	yes	yes	yes	yes	yes	28	Excellent
Junge et al. (2006) [43]	yes	yes	no	dk	yes	yes	yes	yes	no	no	yes	yes	yes	yes	yes	23	Excellent
Langevoort et al. (2006) [30]	yes	yes	no	dk	yes	yes	yes	yes	no	no	yes	yes	yes	yes	yes	23	Excellent
Luig et al. (2018) [1]	yes	yes	no	dk	yes	yes	yes	yes	no	yes	yes	yes	yes	yes	yes	25	Excellent
Moller et al. (2012) [3]	yes	yes	no	yes	yes	yes	yes	yes	yes	yes	yes	yes	yes	yes	yes	28	Excellent
Mónaco et al. (2013) [38]	yes	yes	no	yes	yes	yes	no	yes	no	yes	yes	yes	yes	yes	yes	24	Excellent
Mónaco et al. (2019) [20]	yes	yes	yes	yes	yes	yes	yes	yes	no	yes	yes	yes	yes	yes	yes	28	Excellent
Olsen et al. (2006) [21]	yes	yes	no	dk	yes	yes	yes	yes	no	dk	yes	yes	yes	yes	yes	24	Excellent
Petersen et al. (2005) [39]	yes	yes	yes	yes	yes	yes	yes	yes	yes	no	no	yes	yes	yes	yes	28	Excellent
Piry et al. (2011) [40]	yes	yes	no	dk	yes	yes	yes	yes	no	no	yes	yes	yes	yes	yes	23	Excellent
Rafnsson et al. (2017) [23]	yes	yes	yes	dk	yes	yes	yes	yes	no	yes	yes	yes	yes	yes	yes	27	Excellent
Tabben et al. (2019) [41]	yes	yes	yes	dk	yes	yes	yes	yes	no	no	yes	yes	dk	yes	yes	24	Excellent
Wedderkopp et al. (1999) [42]	yes	yes	no	no	yes	yes	no	yes	no	yes	yes	yes	yes	yes	yes	22	Good

Two points were awarded for yes; 1 point for don’t know (dk); and 0 points for no.
